# Acute oral toxicity and antioxidant studies of an amine-based diselenide

**DOI:** 10.1186/s12906-019-2489-5

**Published:** 2019-04-03

**Authors:** Mohammad Ibrahim, Niaz Muhammad, Musadiq Ibrahim, Muhammad Idrees Khan, Muhammad Ishaq Ali Shah, Muhammad Said, Waliullah Khan, Jean Paul Kamdem, Joao Batista Teixeira Rocha

**Affiliations:** 1 Department of Chemistry, Abdul Wali Khan University Mardan (AWKUM), KPK, Mardan, Pakistan; 2 Departamento de Química, Centro de Ciências Naturais e Exatas, Universidade Federal de Santa Maria, Santa Maria, RS CEP 97105-900 Brazil; 3 Department of Chemistry and Division of Biochemistry, Life Science University of Glasgow, Glasgow, G128QQ UK; 4 Department of Chemistry, Kohat University of Science and Technology, Kohat, Khyber Pakhtun Khwa Pakistan; 5 Department of Biological Sciences, Regional University of Cariri Campus Pimenta, Crato-CE, Ceará CEP 63105-000 Brazil

**Keywords:** Organoselenium, LD_50_, Toxicity, Lipid peroxidation, Antioxidant, Vitamin C

## Abstract

**Background:**

Organochalcogen compounds have attracted the interest of a multitude of studies for their promising Pharmacological and biological activities. The antioxidant activity and acute toxicity of an organoselenium compound, 1-(2-(2-(2-(1-aminoethyl)phenyl)diselanyl)phenyl)ethanamine (APDP) was determined in mice.

**Methods:**

Mice were randomly divided into four groups, with each group comprising of seven animals. Canola oil (1ml/kg of body weight) was administered to 1st group, while 2nd, 3rd & 4th groups were administered with 10 mg/kg, 30 mg/kg & 350 mg/kg of APDP respectively. APDP was administered by Intragastric gavage as a single oral dose.

**Results:**

The APDP oral administration was found to be safe up to 350 mg/kg of body weight and no deaths of animals were recorded. The lethal dose 50 (LD_50_) for APDP was determined at 72 h and was estimated to be > 350 mg/kg. After acute treatment, all mice were sacrificed by decapitation to determine the antioxidant enzymes and lipid peroxidation values for the treated mice liver. No fluctuation in lipid peroxidation, vitamin C and non protein thiol (NPSH) levels was observed due to the administration of APDP. hepatic α-ALA-D activity, catalase (CAT), superoxide dismutase (SOD) and the biochemical parameters were evaluated. Experimental observation demonstrated that APDP protected Fe(II) induced thiobarbituric acid reactive substances (TBARS) production in liver homogenate significantly (*p* < 0.05). The administration of APDP (an amine-based diselenide) both in vitro and in vivo clearly demonstrated that this potential compound has no acute toxicity towards mice among all the tested parameter.

**Conclusion:**

On the basis of experimental results, it is concluded that APDP is a potential candidate as an antioxidant compound for studying pharmacological properties.

## Background

A dynamic equilibrium exists between the antioxidant capacity of the cell and the generation of the reactive oxygen species (ROS) in the normal conditions, which is critical for the survival as well as normal functioning of the aerobic organisms. Subsequently, an imbalance of the equilibrium potentially leads to damages, known as oxidative stress [1]. In the pathogenesis of various diseases and the process of accelerated aging, different biochemical, biological, and clinical studies depict the involvement of oxidative stress induced by reactive oxygen species (ROS) [2]. To cope with this phenomenon, aerobic organisms have evolved a potent antioxidant network in which many antioxidants play their respective role to avoid oxidative stress [3]. These include enzymes, proteins & some bio-organic compounds such as catalase (CAT), glutathione peroxidase (GPx), superoxide dismutase (SOD), ferritin, ceruloplasmin, glutathione, vitamin C, and vitamin E. Due to their beneficial role in terms of antioxidant activity, maintenance of human health, prevention and treatment of degenerative diseases, these biological compounds have gained considerable attention from scientists, physicians, and the general public [4].

Recently, synthetic antioxidant compounds have been making their swift march but medicinal chemists have now realized that the discovery of mere exogenous antioxidants is insufficient. A vital need exists for novel drug that express antioxidant activity, and essentially act through clinically unexploited mode of action. Selenium serves as a micronutrient in humans, its deficiency can potentially lead to a number of viral infections causing neurodegenerative disorders, cardiovascular issues, arthritis, cancer and cataracts [5]. Even though the mechanistic background of selenium toxicity is still not clearly understood. Consequently, toxicity is attributed to the ability of the seleno-compounds to generate reactive oxygen species (ROS) and to induce oxidative stress. Furthermore, various symptoms of the selenium toxicity include nail discoloration, nausea, vomiting,, loss of hair, fatigue, brittleness irritability, and foul breath odor (often described as garlic breath) [5].

Organoselenium compounds have attracted scientific community in general and medicinal chemists in specific due to their potent pharmacological activities. APDP is a synthetic based simple organoselenium compound, widely reported to display anti-inflammatory, antioxidant, and neuroprotective effects in disease models and several chemically-induced toxicity [6, 7]. In addition, the antioxidant activity of APDP has been associated with its ability to mimic glutathione peroxidase [5]. In another study APDP provide neuroprotection against 6-hydroxydopamine (6-OHDA) toxicity in differentiated human SH-SY5Y cells [8]. In this regard, our research group has recently reported the ROS-scavenging activity of a simple, organo-selenium compound, binapthyl diselenide (NapSe)_2_ [9]. Its antioxidant activity has been experimentally established by its ability to protect mice brain from free radical induced oxidative stress, both in vitro and in vivo [10]. Furthermore, organoselenium compounds have been reported to have a protective role in a variety of experimental models associated with the overproduction of free radicals [5]. These promising reports prompted us to hypothesize that 1-(2-(2-(2-(1-aminoethyl)phenyl)diselanyl)phenyl)ethanamine (APDP) may have a role in combating the oxidative stress.

Therefore the study was carried out on mice to evaluate the effects of APDP in protecting Fe(II)-induced oxidative stress in case of acute liver toxicity (in vivo) and liver homogenate (in vitro).

## Methods

### Chemicals

**Fig. 1:**
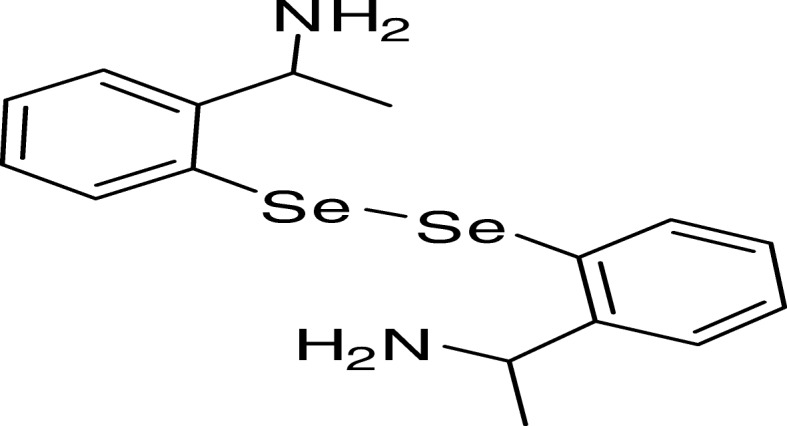
1-(2-(2-(2-(1-aminoethyl)phenyl)diselanyl)phenyl)ethanamine (APDP) chemical structure

1-(2-(2-(2-(1-aminoethyl)phenyl)diselanyl)phenyl)ethanamine (APDP) (Fig. 1) was synthesized according to literature methods [10], with little modifications and was dissolved in DMSO. Purity of the compound was checked by ^1^H NMR and ^13^C NMR spectra and was found 99.9% pure. Furthermore, spectroscopic and analytical data was in full agreement with the assigned structure. All other chemicals were purchased from Sigma®.

### Experimental animals

Adult male Swiss mice (25–35 g) from breeding colony. Two mice were kept in each cage, on a 12-h/12-h light/dark cycles, respectively, within a temperature range of 22–24°C around 56% humidity level and an easy approach to food and water. The animals were screened for in vitro and in vivo experiment following ethical codes of the Animal Care and Wellness Committee of the Universidade Federal de Santa Maria RS, Brazil (23,081.002435/2007–16).

### In vitro experiments

The in vitro TBARS assay was performed to evaluate antioxidant effect of APDP.

### TBARS) assay

#### Preparation of tissue homogenate

The Mice were subjected to mild ether anesthesia followed by decapitation. The liver was rapidly dissected, placed on ice and weighed. Liver tissues were instantly homogenized in cold 10mM Tris–HCl, pH 7.5 (1/10, *w*/*v*) at around 1200 rev/min in a Teflon-glass homogenizer. The homogenate was centrifuged for 10 min at 4000×*g* to produce a pellet and a low-speed supernatant (S1). An aliquot of 100 μl of S1 was incubated for 1 h at 37 °C in the presence of both organodiselenide with and without the prooxidants (iron final concentration 10μM). The incubated sample (S1) was then subjected to lipid peroxidation assay examination. TBARS formation was determined as reported by Ohkawa [10] . The reaction was started by the addition of 8.1% sodium dodecyl sulphate (SDS) to S1, followed by successive mixing of 500μl acetic acid/HCl (pH 3.4) and 0.8% thiobarbituric acid (TBA). The reaction mixture was then incubated at 95 °C for 1 h. TBARS production was monitored at 532 nm and compared to malondialdehyde (MDA) absorbance, used as a standard.

### Ex vivo experiments

#### Lethality

Four groups of Mice each containing randomly selected seven animals were made. First group was given canola oil (1ml/kg of body mass). Second, third and fourth groups were given 10 mg, 30 mg and 350 mg of APDP (per kg of body mass) respectively. A single oral dose of APDP was given by intragastric gavage. The lethal dose 50 (LD_50_) was determined 72 h after APDP administration.

#### Aspartate aminotransferase (AST) and alanine aminotransferase (ALT) activities

As a biochemical marker for the early acute hepatic damage, Aspartate aminotransferase (AST) and Alanine aminotransferase (ALT) activities were performed as per colorimetric procedure reported by Reitman and Frankel [11], using commercial kits (LABTEST, Diagnostic S.A. Minas Gerais, Brazil).

#### Lipid peroxidation

Thiobarbituric acid-reactive species (TBARS) assay reported by Ohkawa was used for the low-speed supernatant (S1) of liver [10]. The amount of TBARS produced was measured at 532 nm, after incubating sample for 2 h at 95°C against an external standard, MDA.

#### Catalase activity

Catalase assay was performed by following the procedure as reported earlier [12] by monitoring the disappearance of hydrogen peroxide in the presence of low-speed supernatant (S1) at 240 nm. The enzymatic reaction was started by the addition of an aliquot of 20 μl of S1 and 0.3 mM substrate (H2O2) to the reaction medium containing 50 mM phosphate buffer of pH 7.0.

#### Superoxide dismutase (SOD)

A spectrophotometric method based on the ability of SOD to inhibit the autoxidation of epinephrine to adrenochrome was used to perform SOD assay at 480 nm [13]. Aliquots of a 1:10 (*v*/v) dilution of S1 were added in a 50-mM glycine buffer of pH 10.3, followed by the addition of epinephrine to start the enzymatic reaction. One unit of enzyme was defined as the amount of enzyme required to inhibit the rate of epinephrine autoxidation by 50% at 26 °C. The enzymatic activity was expressed as units per milligram protein.

#### Non-protein thiols (NPSH)

Ellman method was performed to estimate NPSH [14]. Two hundred microliter of 10% trichloroacetic acid was added followed by centrifugation to precipitate protein fraction. 1M phosphate buffer at pH 7.4 and 10 mM DTNB were used for the experiment at 412 nm.

#### Vitamin C levels

Rocha method with slight changes was used to find Vitamin C level [15]. The low-speed supernatant (S1) was precipitated in 10% trichloroacetic acid solution. A 300 μl of the sample was incubated for 180 min at 37 °C followed by the addition of 500 μl H2SO4 65% (*v*/v) to the reaction mixture. Dinitrophenyl hydrazine (4.5 mg/ml) and copper(II) sulphate (0.075 mg/ml) were used to estimate the reaction product at 520 nm.

#### δ-ALA-D activity

Sassa method for the determination of δ-ALA-D activity was used [16] by measuring the rate of product of porphobilinogen (PBG) formation. Erlich’s reagent at 555 nm was used to determine the reaction product.

#### Protein determination

Protein concentration was determined by the Bradford method [17] against bovine serum albumin as a refrence.

#### Statistical analysis

Results were analysed by applying a one-way analysis of variance (ANOVA) for APDP toxicity experiment. Duncan’s Multiple Range Test was used when appropriate. Main effects are presented only when the second order interaction was non-significant. All data were expressed as means ± S.D. *p* < 0.05 were considered statistically significant.

## Results

### TBARS results in vitro experiments

**Fig. 2:**
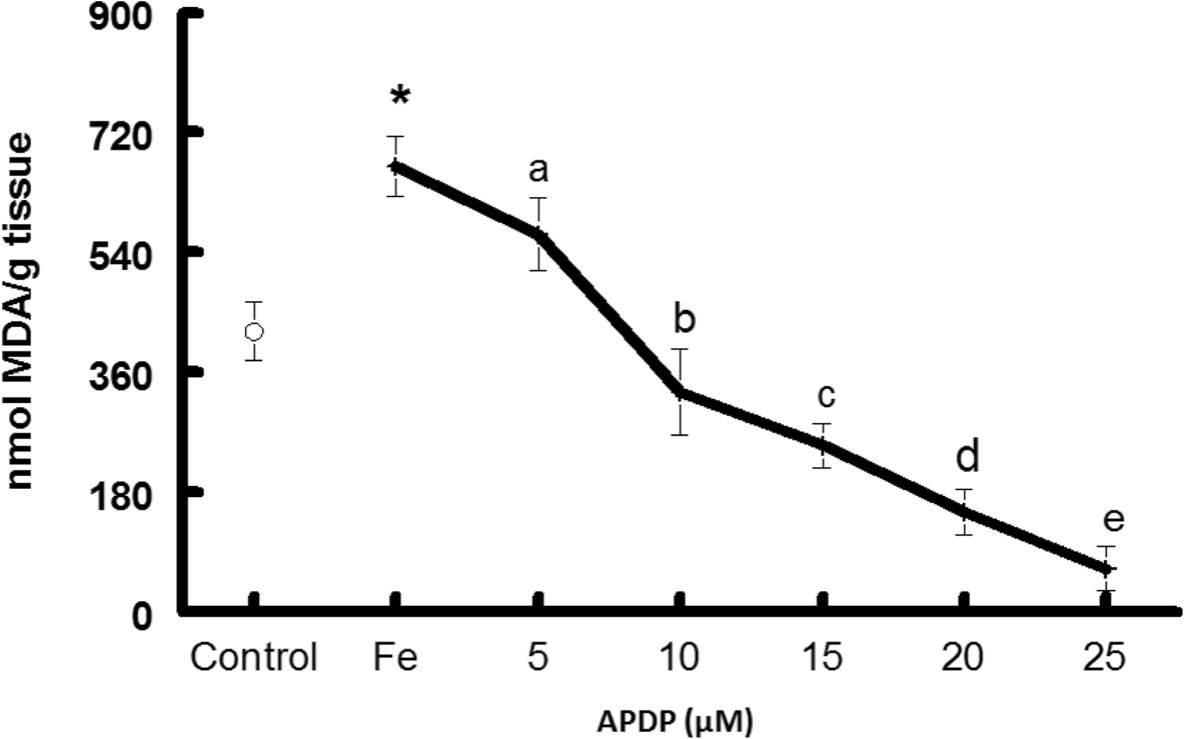
Effect of 1-(2-(2-(2-(1-aminoethyl)phenyl)diselanyl)phenyl)ethanamine (APDP) on lipid peroxidation induced by 10 μM iron (II)-induced in liver homogenates. Data show mean ± SD values averaged from four independent experiments performed in quadruplicate in different days Different letters indicate a significant difference in relation to the control (without pro-oxidant) and among the groups (with pro-oxidant) at *p* < 0.05. Asterisk shows significant effect of iron (II) at p < 0.05, Abbreviations: Fe—Iron

In TBARS experiment Iron(II) sulphate triggered a noteworthy increase in TBARS generation in mice liver homogenates (Fig. 2). When these Fe(II) induced TBARS were challenged with APDP caused a significant (*P* < 0.05) reduction in Fe(II)-induced TBARS generation in liver homogenate (Fig. 2), indicating the antioxidant effect by lowering the Fe(II) effect in mice homogenate.

### Lethal dose _50_

Exposures of mice to APDP have shown no mortality at all tested doses. The determined LD_50_ was found to be > 350 mg/kg.

### Lipid peroxidation

It was observed that all the three doses of the tested compound, APDP did not change the TBARS level which means this compound have no toxic effect on the lipid (Table1).

### δ-ALA-D assay

Acute treatment with APDP have shown no changes in Hepatic δ-ALA-D activity as shown in the Table 1.

**Table 1:** Effect of 1-(2-(2-(2-(1-aminoethyl)phenyl)diselanyl)phenyl)ethanamine (APDP) on hepatic TBARS (Thiobarbituric acid reactive substances), NPSH (Non-protein thiol) and δ-ALA-D (Delta-aminolevulinic acid dehydratase) of mice

Groups(mg/kg)	TBARS^a^	NPSH ^b^	δ-ALA-D^c^
Control	28.3 ± 1.2	8.3 ± 0.4	25.3 ± 3.4
APDP-10	30.2 ± 11.1	8.5 ± 2.0	23.5 ± 2.3
APDP-100	29.3 ± 2.1	8.2 ± 2.1	26.3 ± 1.5
APDP-350	33.1 ± 2.1	9.1 ± 2.9	25.2 ± 1.6

Data are reported as mean ± S.D. Data are reported as mean ± SD of seven animals per group. (*) Denoted *p* < 0.05 as compared to all groups (one-way ANOVA/Duncan). Data are expressed as ^a^nmol equivalents MDA (malondialdehyde) mg-1 protein, ^b^μg ascorbic acid g-1 tissue, ^c^U mg-1 protein and nmol of porphobilinogen mg-1 protein hour-1.

### NPSH levels

NPSH levels have shown no significant changes among the tested groups compared to the control group (Table 1).

### AST and ALT activites

No significant changes were observed in plasmatic activities AST and ALT among the three groups (Table 2) after exposure to different doses (10, 100 and 350 mg/kg) of APDP.

**Table 2:** Effect of 1-(2-(2-(2-(1-aminoethyl)phenyl)diselanyl)phenyl)ethanamine (APDP) on ALT (alanine transaminase) and AST (aspartate transaminase) in mice

Groups(mg/kg)	ALT	AST
Control	52.2 ± 4.1	33.2 ± 2.3
APDP-10	54.3 ± 11.3	31.0 ± 11.1
APDP-100	51.2 ± 3.2	34.0 ± 4.3
APDP-350	50.1 ± 12.2	30.2 ± 5.7

Data are reported as mean ± S.D. Data are reported as mean ± SD of seven animals per group. (*) Denoted *p* < 0.05 as compared to all groups (one-way ANOVA/Duncan). AST and ALT are expressed as U/ml

### Catalase activity

Hepatic catalase activity was not altered with different doses (10, 100 and 350 mg/kg) of APDP when compared to the control group (Table 3).

**Table 3:** Effect of 1-(2-(2-(2-(1-aminoethyl)phenyl)diselanyl)phenyl)ethanamine (APDP) on hepatic SOD (superoxide dismutase), CAT (catalase) and Ascorbic Acid of mice

Groups(mg/kg)	SOD^a^	CAT^b^	Ascorbic Acid^c^
Control	21 ± 4.2	65.7 ± 10.7	70.2 ± 3.2
APDP-10	19 ± 5.3	122.6 ± 50.4	72.1 ± 15.6
APDP-100	23 ± 6.1	57.2 ± 10.3	75.4 ± 7.3
APDP-350	18 ± 5.2	53.0 ± 8.9	76.5 ± 4.0

Data are reported as mean ± S.D. Data are reported as mean ± SD of seven animals per group. (*) Denoted *p* < 0.05 as compared to all groups (one-way ANOVA/Duncan).

Data are expressed as ^a^U SOD mg-1, ^b^U mg-1 protein, ^c^μg ascorbic acid g-1 tissue

### Superoxide dismutase

APDP did not alter SOD with different doses of (10, 100 and 350 mg/kg), APDP when compared to the control group (Table 3).

### Ascorbic acid

APDP did not change ascorbic acid level with different doses (10, 100 and 350 mg/kg) of APDP when compared to the control group (Table 3).

### Vitamin C levels

Exposure to APDP did not alter hepatic levels of vitamin C (Table 3).

## Discussion

The studies described herein, therefore, investigated in vitro and in vivo toxicity of an amine-based diselenide and examined the possible protective effects of Se against Fe(II)- induced TBARS [7]. According to a survey of the literature, the organoselenium are potent and have interesting biological properties than the inorganic selenium [6].

It is well established that organoselenium compounds are superior drugs than other sulfur analogue in and have many biological functions, especially in the antioxidant defense system [7].

It was confirmed from in vitro experiment that Fe(II)-TBARS formation significantly protected by APDP in mice liver homogenate, demonstrating strong antioxidant activity (IC_50_ about 10μM) (Fig. 2). Based on our previous results, we performed in vivo study to find out the acute oral toxicity study of amine-based diselenide 1-(2-(2-(2-(1-aminoethyl)phenyl)diselanyl)phenyl)ethanamine in mice at different doses (10 mg/kg, 100 mg/kg and 350 mg/kg).

Exposure of mice to a single dose of APDP did not produce any treatment-related effects. No remarkable changes were observed in serum biochemistry after acute exposure of APDP. The LD_50_ for the oral administration of APDP was estimated to be > 350 mg/kg. Therefore, it is safe to state that, APDP a diselenide can be considered a drug which causes low toxicity. (Tables 1, 2 and 3).

Our earlier publications related to the acute toxicity effects of diphenyl diselenide and binapthyl diselenide have reported that these compounds were nontoxic and showed promising pharmacological properties [18].

APDP meaningfully reduced iron(II)-stimulated TBARS in liver tissues (Fig. 2). This encouraged us to evaluate APDP acute toxicity test in mice. Here it is important to mention that amine-based diselenide is capable of non-bonded nitrogen interactions which we believe is responsible for its low IC_50_ and higher antioxidant properties in vitro results.

In the present study mice exposed to APDP did not change markers of hepatic function and showed no significant difference with control group. In our previous study, we demonstrated that liver and kidney profiles were not altered with acute treatment of binapthyl diselenide. In another study from our group demonstrated that rabbits chronical exposure to diphenyl diselenide enriched food has not affected the normal hepatic and renal functions [19].

δ-ALA-D is a toxicity biomarker for various xenobiotics [20]. In this study the organoselenium compound APDP has shown no change in δ-ALA-D activity which further confirmed its non-toxic nature (Table 1). Some authors reported that organoseleniums can show interaction with δ-ALA-D -SH moieties [21, 22]. Similar to present results, in our previous work we reported that organoselenium compound did not modify δ-ALA-D activity in mice.

The levels of TBARS, a marker of lipid peroxidation, were not changed after oral exposure to APDP in the liver, kidney or brain of the treated mice compared to the control group (Table 1). The data suggest that this compound did not cause oxidative stress in mouse tissue following acute treatment for 72h. These results are in agreement with the findings of other researchers of our group.

Our experimental study demonstrates that, SOD (one of the first antioxidant enzymes in the line of defence against the deleterious effects of oxygen radicals in the cells, scavenges ROS by catalyzing the dismutation of superoxide to hydrogen peroxide (H_2_O_2_) and CAT (critical for ROS detoxification when high levels of H_2_O_2_ are produced) [23], activities were not changed significantly at all dosages of APDP compared with the control group (Table 3).

## Conclusions

By analyzing all the experimental data, we concluded that APDP did not alter lipid peroxidation, vitamin C, non NPSH, hepatic α-ALA-D activity, CAT and SOD activity even at higher doses i.e 350 mg/kg of body weight of mice and also showed excellent antioxidant activities against Fe(II) induce lipid peroxidation in mice liver homogenate.

On the basis of experimental results, it is concluded that APDP is a potential candidate as an antioxidant compound for studying pharmacological properties.

## Abbreviations

(NapSe)_2_: Binapthyl diselenide
ALT: Alanine aminotransferase
ANOVA: One-way analysis of variance
APDP: 1-(2-(2-(2-(1-aminoethyl)phenyl)diselanyl)phenyl)ethanamine
AST: Aspartate aminotransferase
CAT: Catalase
GPx: Glutathione peroxidase
LD_50_: Lethal dose 50
NPSH: Non protein thiol
ROS: Reactive oxygen species
SOD: Dismutase
TBARS: Thio Barbituric Acid Reactive Species
